# Solid-in-Oil Nanodispersions for Transcutaneous Immunotherapy of Japanese Cedar Pollinosis

**DOI:** 10.3390/pharmaceutics12030240

**Published:** 2020-03-07

**Authors:** Qingliang Kong, Momoko Kitaoka, Rie Wakabayashi, Yoshiro Tahara, Noriho Kamiya, Masahiro Goto

**Affiliations:** 1 Department of Applied Chemistry, Graduate School of Engineering, Kyushu University, Fukuoka 819-0395, Japan; kong.qingliang.445@s.kyushu-u.ac.jp (Q.K.); mkitaoka@mail.cstm.kyushu-u.ac.jp (M.K.); rie_wakaba@mail.cstm.kyushu-u.ac.jp (R.W.); ytahara@mail.doshisha.ac.jp (Y.T.); kamiya.noriho.367@m.kyushu-u.ac.jp (N.K.); 2 Advanced Transdermal Drug Delivery System Center, Kyushu University, Fukuoka 819-0395, Japan; 3 Center for Future Chemistry, Kyushu University, Fukuoka 819-0395, Japan

**Keywords:** allergen-specific immunotherapy, Japanese cedar pollinosis, solid-in-oil nanodispersions, transcutaneous immunotherapy

## Abstract

Japanese cedar pollinosis (JCP) is a common affliction caused by an allergic reaction to cedar pollen and is considered a disease of national importance in Japan. Antigen-specific immunotherapy (AIT) is the only available curative treatment for JCP. However, low compliance and persistence have been reported among patients subcutaneously or sublingually administered AIT comprising a conventional antigen derived from a pollen extract. To address these issues, many research studies have focused on developing a safer, simpler, and more effective AIT for JCP. Here, we review the novel antigens that have been developed for JCP AIT, discuss their different administration routes, and present the effects of anti-allergy treatment. Then, we describe a new form of AIT called transcutaneous immunotherapy (TCIT) and its solid-in-oil (S/O) nanodispersion formulation, which is a promising antigen delivery system. Finally, we discuss the applications of S/O nanodispersions for JCP TCIT. In this context, we predict that TCIT delivery by using a S/O nanodispersion loaded with novel antigens may offer an easier, safer, and more effective treatment option for JCP patients.

## 1. Introduction

Japanese cedar *(Cryptomeria japonica*, JC) pollinosis (JCP) is a type I allergic rhinitis caused by an overreaction to cedar pollen. It affects around 30% of people in Japan, with impacts on daily activity and work productivity, and its prevalence has increased 2.6-fold from 1980 to 2000 [1]. It has already become a major medical and public health issue in Japan and, therefore, is considered a disease of national importance in Japan [2]. Allergen avoidance, pharmacotherapy, and allergen-specific immunotherapy (AIT) are currently the main options for JCP patients. However, patients cannot completely avoid JC pollen by wearing a mask and glasses. Most patients receive pharmacotherapies, including oral or intranasal administration of antihistamines and intranasal administration of aerosol corticosteroids, to relieve the symptoms of JCP [2]. AIT is the only curative treatment for JCP. It involves repeatedly administering the JC allergen to a patient with increasing doses to induce antigen-specific immune tolerance and, thus, reduce the reliance on pharmacotherapy [3,4].

Subcutaneous immunotherapy (SCIT) with JC pollen extract (PE) is a traditional form of AIT that was introduced as a clinical treatment 100 years ago. To induce a long-term tolerance, SCIT must be administered for up to 3–5 years, escalating gradually from a low PE dose in the initial phase to a high dose in the maintenance phase. Although SCIT is more effective than pharmacotherapy, severe allergic side effects, and even death, have been reported after injection of JC PE. In addition, patients find it difficult to endure the pain of the weekly injection over the long therapeutic period. Therefore, only 18% of patients finish a 3 year treatment course [5].

Sublingual immunotherapy (SLIT) was introduced as a simple, convenient alternative to SCIT because the standard allergen PE is administered at home as tablets or drops rather than an injection. JCP SLIT treatment has already been approved by regulatory authorities in Japan. Although no severe allergic side effects caused by SLIT have been reported, other side effects, including itching and swelling of the mouth and tongue, headache, and ear pruritus, have been observed [6]. Moreover, in clinical practice, poor adherence to both the SCIT and SLIT treatment courses has been reported [5]. This low compliance may be attributed to the long therapeutic period of up to 3-5 years to achieve the therapeutic effect. In addition, the cost of a long therapeutic period may also limit the broad applicability of SCIT and SLIT. Therefore, there is a need for a safe, simple, and inexpensive immunotherapy for JCP that can rapidly induce JC allergen-specific immune tolerance.

Several novel therapies with different administration routes are being developed as alternatives to SCIT and SLIT for JCP immunotherapy [3]. Oral immunotherapy (OIT) attracts the most attention in JCP AIT research because it is also commonly used for treating food allergies. A relatively large amount of allergen is orally delivered to immune cells in the gut that are associated with lymphoid tissue, thus effectively inducing immune tolerance. However, a few other alternative methods have been proposed such as transcutaneous immunotherapy (TCIT) and epicutaneous immunotherapy (EPIT). TCIT/EPIT involves continuous administration of the antigen through the skin to target several antigen-presenting cells (APCs) found in the skin to promote the immune tolerance and mitigate both local and systemic adverse effects [7,8]. Successful phase 3 trials of EPIT for peanut allergy suggest the potential for applying TCIT/EPIT to JCP AIT [9].

In this article, we review the published research studies of advanced AIT methods for JCP. We summarize the novel antigens that have been tested for JCP AIT, including the use of T cell epitope peptides derived from the JC allergen, modified allergens derived from the JC allergen, DNA vaccines encoding either the JC allergen gene or the T cell epitope peptide gene, and adjuvant conjugations. Although several administration routes for JCP AIT have been investigated, few research studies have focused on TCIT for JCP. Therefore, we further introduce a novel TCIT using solid-in-oil (S/O) nanodispersions, which we predict will be effective against JCP.

## 2. Overview of Antigens Developed for Antigen-specific Immunotherapy (AIT) of Japanese Cedar Pollinosis (JCP)

The mechanisms of JCP and AIT have been summarized in recent literature [4,10]. Based on this present knowledge about AIT, the most important aspect for effective JCP AIT is avoiding side effects associated with antigens and improving the vaccine efficiency of antigens to shorten the duration of the therapy. To realize these goals, researchers generally focus on the most crucial aspect of AIT: antigens. There is a need for a “safe” antigen that does not cross-link the allergen-specific immunoglobulin E (IgE) antibody. Moreover, possible approaches to shorten the duration of AIT include modifying the allergen used in AIT by, for example, attaching an adjuvant to suppress Th2 immunity or binding polysaccharides or polymers to facilitate uptake through immune cells or target immune cells.

Several antigens have been developed for JCP AIT including T cell epitope peptides, modified allergens, DNA vaccines, and adjuvant conjugations; they are summarized in Table 1. While most research studies are still at the preclinical stage, the phase I clinical trials of OIT with transgenic rice seeds (UMIN000010212, UMIN000011086, UMIN000016078, UMIN-000024699, UMIN-000024700 and UMIN000034280) have already been completed [11], and OIT with a Cry j 1-galactomannan conjugate (UMIN000011995 and UMIN000013408) and intramuscular immunotherapy with a lysosomal-associated membrane protein (LAMP)-based DNA vaccine (NCT03101267) have been tested in the phase II clinical trials. 

### 2.1. Cry j Allergens and PE 

Cry j is a registered allergen derived from *Cryptomeria japonica* [12]. Cry j 1 and Cry j 2 are two major allergens of JC PE and have already been characterized [13,14]; they are described in detail in [12]. Cry j 1 was the first allergen isolated from JC PE; it is a basic protein with a molecular weight (Mw) of 45–51 kDa and exhibits pectate lyase activity. Cry j 2, the second allergen isolated from JC PE, is a protein with a Mw of 37–45 kDa and is a homolog of polygalacturonase. New allergens from JC PE have been reported, such as Cry j 3 and Cry j 4 [15,16]; however, allergens Cry j 1 and Cry j 2 still affect the largest proportion (more than 95%) of JCP patients [17].

Although crude PE has been used as the antigen for JCP SCIT since 1969, standard PEs for SCIT and SLIT were approved in 2000 and 2014, respectively. A unit of standard PE is defined based on the amount of Cry j 1 allergen. A sublingual drop, Cedartolen^®^, and a sublingual tablet, Cedarcure^®^, derived from standard PE (Torii Pharmaceutical Co., Ltd., Tokyo, Japan) were approved and became available on the market in 2014 and 2018, respectively. While the standard PE is effective as an antigen in SCIT and SLIT for the treatment of JCP, it does induce side effects. The complexity of whole allergens in PE may induce IgE-medicated acute side effects in patients. Moreover, the side effects may contribute to the dose limitations and long treatment duration. To resolve these issues, novel antigens have been developed for JCP AIT; they are described as follows.

### 2.2. T Cell Epitope Peptide

**Figure 1:**
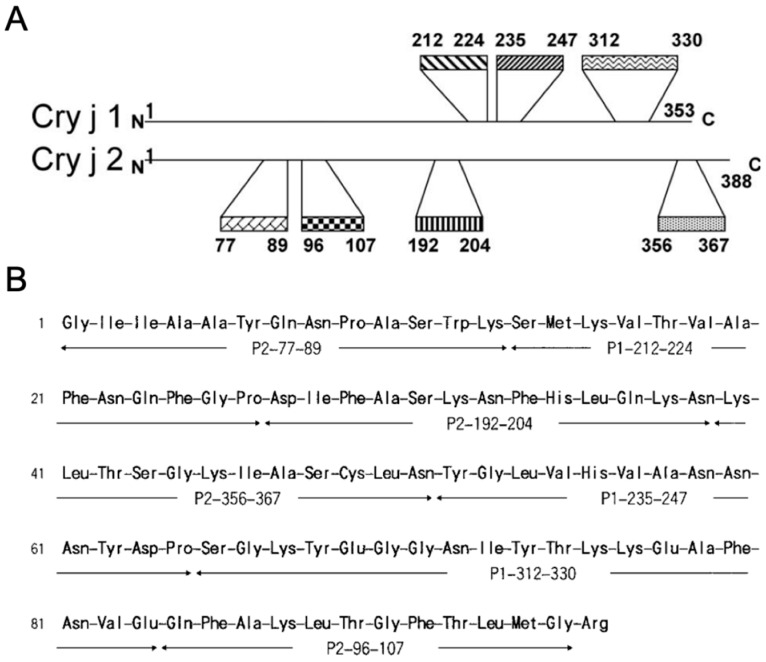
(**A**) Seven major T cell determinants of Cry j 1 and Cry j 2 in patients with Japanese cedar pollinosis. (**B**) Amino acid sequence of the hybrid peptide 7Crp. Reproduced from [50] with permission of Elsevier, 2011, and [49], with permission from Elsevier, 2001.

A T cell epitope derived from pollen allergens has been proposed as an antigen to reduce the risk of side effects caused by the use of whole PE [18]. The T cell epitope is a short allergen-derived peptide recognized by the T cell receptor. It does not cross-link the allergen-specific IgE bound to the surfaces of the effector cells and, therefore, does not induce side effects. Several dominant T cell determinants have already been identified as important T cell epitope peptides in patients with JCP (Figure 1A) [17,19].

The T cell epitope peptide p246-259 (RAEVSYVHVNGAKF) derived from allergen Cry j 2 is an immunodominant T cell determinant in BALB/c mice. OIT with the p246–259 has been shown to inhibit allergen-specific Th1 and Th2 cell responses [20]. The orally administered peptide induced tolerance in Cry j 2-sensitized mice via T cell responses against the JC allergen. The mice exhibited reduced levels of allergen-specific Th1- and Th2-mediated antibodies and cytokines, including Th1-mediated antibodies IgG2a, IgG2b, cytokines IFN-γ and IL-2, Th2-mediated antibodies IgG1, IgE and cytokine IL-4. A later report showed that OIT with the p246–259 significantly suppresses the allergic symptom of sneezing frequency in Cry j 2-sensitized mice, indicating that the oral administration of p246–259 is a promising approach for JCP immunotherapy [21]. This is the only single epitope peptide that has been evaluated in JCP-sensitized mice. However, humans have different genetic backgrounds and may respond differently to T cell epitope peptides, so a single T cell epitope may not be sufficient to treat all patients [48].

A recombinant hybrid peptide comprising several T cell epitopes is another approach for JCP AIT that could overcome the drawbacks of a single epitope. Several hybrid peptides comprising different T cell epitopes were developed, including integrated peptides from three T cell epitopes [22], Cry-consensus from five or six T cell epitopes [17,26], 7Crp from seven T cell epitopes [49], and hybrid peptides from fourteen T cell epitopes [23]. The most well-known recombinant hybrid peptide is 7Crp, which comprises three T cell epitope peptides of Cry j 1 and four epitope peptides of Cry j 2 (Figure 1B). Hirahara et al. reported that 7Crp induces the T cell proliferative response as well as Cry j 1 and Cry j 2 allergens. Therefore, it is expected that 7Crp would downregulate JC allergen-specific T cells as effectively as Cry j 1 and Cry j 2 [49]. 

Takagi et al. developed a transgenic rice seed in which 7Crp is expressed and accumulates in the rice seed as the antigen for JCP OIT [24]. Both the T cell proliferative response to Cry j 1 allergen and the IgE serum antibody level were reduced after rice seeds containing 7Crp were orally administered to Cry j 1-sensitized mice. These results suggest that OIT with transgenic rice seeds containing 7Crp could induce immune tolerance to Cry j 1. Several clinical trials of OIT with transgenic rice seeds containing 7Crp have already been completed (UMIN000010212, UMIN000011086, UMIN000016078, UMIN-000024699, UMIN-000024700 and UMIN000034280). No adverse events occurred in the clinical studies, however, the efficacy of OIT using transgenic rice on allergic symptoms was not recognized in 48-week trail due to the limited design of these clinical studies (UMIN-000024699 and UMIN-000024700) [11]. Similarly, Kawabe et al. generated genetically-manipulated chickens expressing a fusion protein of chicken egg white lysozyme and 7Crp [25]. OIT with 7Crp-containing egg white in allergic mice significantly reduced the total and Cry j 1-specific IgE levels compared with a diet including normal egg whites. One of the clinical symptoms of JCP, the number of sneezes, was suppressed in allergic mice fed with the 7Crp-containing egg white. These results indicate that OIT with 7Crp-containing rice seed or egg white is effective for JCP immunotherapy. In addition to these OIT techniques with rice seeds or chicken eggs containing 7Crp, Yamanaka et al. investigated the use of 7Crp in SLIT for JCP. An IL-10–producing regulatory T cell (Tr1) was used to evaluate the change of regulatory T cells. Patients undergoing the SLIT of 7Crp showed increased IL-10-producing Tr1 cells, suggesting that SLIT with 7Crp also induces immune tolerance [27].

### 2.3. Modified Allergen

Although AIT using T cell epitope peptides appears to be safe and effective, this treatment is not sufficient for all JCP patients because humans have unique genetic backgrounds, such as genetic differences in T cell receptors, that may result in different responses to the T cell epitope peptides and insufficient responses in some JCP patients [48]. Hence, modified allergens, including whole spectrum of T cell epitopes, are expected to be effective in all JCP patients. The main challenge remaining is preventing the allergen from cross-linking of the allergen-specific IgE bound to the surfaces of the effector cells.

One approach is attaching a polysaccharide or polymer to the allergen to mask the IgE-binding epitopes in the allergen. One group successfully attached a polysaccharide galactomannan (GM), to mask the IgE-binding epitopes in Cry j 1 [51]. The binding of the IgE from JCP patients to allergen Cry j 1 was completely inhibited by conjugation with GM [52]. The binding of GM to Cry j 1 was also proven to effectively enhance the uptake of antigens into gut dendritic cells, suppressing the Th2 response and reducing the risk of anaphylaxis. Several clinical trials have already proven the safety and efficacy of OIT with the Cry j 1-GM antigen for treating JCP [30,31,32,33]. Compared with the control group, the symptom–medication scores were significantly improved in patients who received OIT with Cry j 1-GM, and the production of allergen-specific IgG4 and IL-10 in peripheral blood mononuclear cells was elevated. Furthermore, no severe side effects were observed in the patients receiving OIT with the Cry j 1-GM antigen. Fujimura et al. developed a fusion protein comprising Cry j 1 and Cry j 2 (Cry j 1/2) modified by polyethylene glycol (PEG) [35]. To prevent cross-linking of the allergen and allergen-specific IgE, the conformational structure of fusion protein Cry j 1/2 was broken by replacing cysteine residues with serine residues and conjugating the modified protein with PEG. Results showed that the level of Cry j 1-specific IgE was significantly reduced and the level of IgG was significantly increased following PEG-Cry j 1/2 treatment in mice and monkeys. Moreover, the Th2-type cytokine levels were suppressed by the PEG-Cry j 1/2 treatment in mice [35].

Another approach to prevent the specific IgE binding is to deconstruct the allergen and use a fusion protein comprising the fragment. Wakasa et al. developed a fusion protein derived from three peptide fragments of Cry j 1 and a deconstructed Cry j 2 protein [36]. This fusion protein was deposited in transgenic rice seeds. Significant decreases in allergen-specific CD4+ T cell proliferation, IgE and IgG antibody levels, and sneezing frequency were observed in mice given OIT with the transgenic seeds compared with those given normal rice seeds. Therefore, OIT with transgenic seeds was considered to induce mucosal immune tolerance. However, a remaining challenge is that OIT requires 100-fold more antigen than SCIT because of the digestive enzymes and harsh conditions in the gastrointestinal tract. In a more recent study, a novel concentrated rice seed containing >70% fusion protein was developed and shown to be highly resistant to enzymatic digestion. Moreover, OIT with the concentrated rice seed induced the specific immune tolerance against allergen Cry j 1 and Cry j 2, indicating its potential use as a novel antigen for JCP OIT [37]. This study is expected to be a bridge to start a clinical trial to evaluate the efficacy of transgenic rice seeds containing the fusion protein.

### 2.4. DNA Vaccine

A DNA vaccine is a novel approach for preventing and treating JCP. In this technique, the antigen gene encoded by bacterial plasmid vectors induces long-lasting expression of a specific antigen, resulting in long-term persistence of the immune response [50]. Unlike T cell epitope peptides and modified allergens, however, DNA vaccines have not been studied extensively, perhaps because they are highly complex. Toda et al. developed a DNA vaccine encoding the Cry j 1 gene. The levels of IgE and IgG 1 antibody produced were lower and IgG 2a antibody produced was higher in mice treated with this DNA vaccine by intramuscular injection, indicating that intramuscular administration of the DNA vaccine effectively induces antigen-specific Th1 immune responses [38]. In a later report, Toda et al. designed a novel DNA vaccine encoding a gene for T cell epitope peptide (p247-258) from Cry j 2. The anti-Cry j 2 IgE level was reduced in mice after administering this DNA vaccine, and the IgG2a response was markedly elevated, suggesting that this treatment has the potential to alleviate JCP allergic reactions [39]. Recently, Su et al. found that fusing a DNA plasmid as an antigen to LAMP can enhance the antigen’s immunogenicity. Two LAMP-based DNA vaccines for JCP, Cry j 1-LAMP and Cry j 2-LAMP, encoding Cry j 1 and Cry j 2, respectively, were developed [40]. The IgE titer was reduced, and the levels of IgG2a and IFN-γ were improved in mice immunized with the Cry j 1-LAMP and Cry j 2-LAMP plasmids, suggesting that robust nonallergenic Th1 response was induced. The safety and long-term immunological effects of Cry j 2-LAMP DNA vaccine against JCP were later evaluated in a Phase IA and IB clinical trial [41]. The results suggested that the Cry j 2-LAMP DNA vaccine is safe and effective for JCP AIT. Astellas Pharma Inc. (Tokyo, Japan) has recently finished a Phase II clinical study of the Cry j-LAMP DNA vaccine (ASP-4070) in Japan (NCT03101267).

### 2.5. Adjuvant Conjugation

As JCP is a typical Th2-induced allergy, the conjugation or formulation of a Th1-enhancement adjuvant, such as CpG oligodeoxynucleotide (ODN) [50], or a T-cell tolerance inducing adjuvant with the allergen are promising strategies to improve the efficiency of AIT. Suzuki et al. conjugated the CpG to a T cell epitope peptide in Cry j 2 for the first time [42], and showed that the levels of Cry j 2-specific IgE, IL-4, and IL-5 were significantly decreased in mice treated with the CpG-Cry j 2 epitope peptide conjugate compared with those in other groups. Moreover, CpG-Cry j 2 peptide conjugate was shown to significantly attenuate nasal symptoms compared with a physical mixture of CpG and the epitope peptide, indicating the importance of the conjugation. In a later study, a CpG-Cry j 1 conjugate was developed, and results in pollinosis-model mice showed that the anti-Cry j 1 IgE level decreased while the IgG2a level increased in those given the conjugate, suggesting that CpG activates Th1 immunity by upregulating IgG2a [43]. Another adjuvant, the cholera toxin B (CTB) subunit, has been used to induce oral tolerance against antigens and modulate the Th1-mediated immune response. Hoang et al. fused CTB with ten T cell epitope peptides of Cry j 1 and Cry j 2 [44], and showed that this protein was antigenic against anti-Cry j 1 and anti-Cry j 2 antibodies. In another study, Takagi et al. fused three T cell epitope peptides with CTB and expressed them in rice seeds as a fusion protein [45]. Results showed that the allergen-specific IgE levels and JC-induced clinical symptoms were significantly reduced in the mice fed the fusion protein compared which control mice.

In contrast to the use of a single molecule adjuvant-antigen conjugate, oligomannose-coated liposomes (OMLs) have been shown to induce a strong Th1 immune response against encapsulated antigens. A tumor immunity study demonstrated that the specific uptake of OMLs by immune cells induced a robust Th1 response, resulting in a significant increase in the cytokine IFN-γ level and a decrease in the cytokine IL-4 level [53]. Ishii et al. investigated the effects of OMLs carrying Cry j 1 and found that OMLs carrying Cry j 1 suppress the serum IgE antibody level and the ratio of IL-5/IFN-γ associated with JCP [46]. This inhibitory effect was attributed to a shift from the Th2 response to the Th1 response. However, to the best of our knowledge, no adjuvant conjugations have been tested in JCP AIT clinical trials, possibly because of concerns about the safety of the adjuvants.

## 3. S/O Nanodispersions Developed for Transcutaneous Immunotherapy (TCIT) of JCP

While SCIT and SLIT are effective for the treatment of JCP, low compliance and persistence were reported, which can be attributed to their side effects [5]. Several other administration routes have been developed as alternatives to SCIT and SLIT for JCP immunotherapy. OIT attracts the most attention in research studies of JCP AIT, but few research studies have focused on TCIT for JCP. Hence, in this section, we present a basic background on transcutaneous antigen delivery and discuss S/O nanodispersions as a novel antigen delivery system.

### 3.1. Background of Transcutaneous Antigen Delivery

Transcutaneous antigen delivery is a simple and safe method for the treatment of allergic diseases because it is non-invasive. In addition, this delivery model helps antigens, which comprise proteins and peptides, avoid gastrointestinal absorption and first-pass elimination. The slow antigen release rate associated with transcutaneous delivery facilitates the immediate cessation of treatment in case adverse events occur. Importantly, there is a variety of APCs in the skin, including Langerhans cells in the epidermis and dendritic cells in the dermis [54], which capture antigens delivered into skin and induce an effective immune response or immune tolerance [7,8]. 

The only challenge associated with transcutaneous antigen delivery is the physical barrier caused by the stratum corneum of the outmost layer of skin. The stratum corneum comprises a brick-and-mortar structure of keratinocytes and intercellular lipids, which form a hydrophobic structure that resists most foreign materials. According to the 500 Dalton rule, only a few highly-lipophilic, small-molecule drugs, such as nicotine, menthol, and estradiol, can cross the stratum corneum barrier and permeate the skin [55,56]. Most of the antigens are hydrophilic proteins or peptides, which cannot permeate the skin. Two different strategies to enhance skin permeation have been developed: Physical technologies, including iontophoresis, electroporation, ultrasound, heat, lasers, jet injection, and microneedle injection, effectively facilitate antigen transport by disrupting the stratum corneum [54,57]. However, these methods generally require specialized instruments and are costly. In contrast, chemical technologies are easy to use and less expensive. Chemical enhancers, including alcohols, amides, esters, glycols, fatty acids, surfactants, and terpenes, help the antigen to penetrate the skin by interacting with the proteins in the skin [58]. Some vesicular-based or lipid-based nanocarriers, such as elastic liposomes, solid lipid particles, nanoemulsions, and microemulsions, have been recently developed and used for transcutaneous antigen delivery [59]. However, most developed nanocarriers for transcutaneous antigen delivery are water-based, and few research studies have investigated oil-based nanocarrier systems.

### 3.2. Development and Application of S/O Nanodispersions for Transcutaneous Antigen Delivery

**Figure 2:**
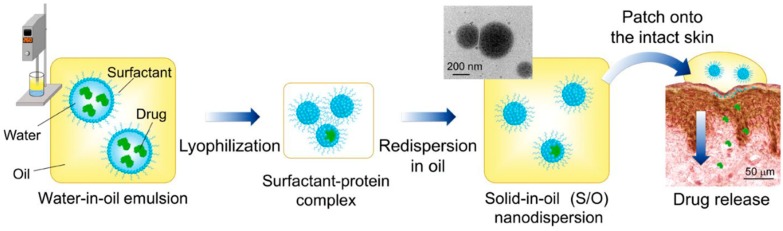
Preparation and application of S/O nanodispersions. Reproduced from [60], which is licensed under a Creative Commons Attribution-(CC BY 4.0) International License.

Considering the lipophobicity of the stratum corneum, we developed an oil-based nanocarrier system referred to as a S/O nanodispersion. S/O nanodispersions are oil-based nano-sized dispersions of an antigen formed by coating antigen molecules with hydrophobic surfactant molecules. The antigen–surfactant complex is derived from a water-in-oil (W/O) emulsion by lyophilization to remove the water and organic solvent followed by dispersion in an oil vehicle (Figure 2) [60]. Compared with a W/O emulsion, S/O nanodispersions exhibit higher antigen-encapsulation efficiency up to 99.5% and better stability of more than 6 months [60,61]. Owing to the hydrophobic surfactant and the oil vehicle, S/O nanodispersions can overcome the barrier of the stratum corneum to deliver antigens into the skin. The main mechanism by which S/O nanodispersions permeate the skin was elucidated by observations using confocal microscopy combined with computational dynamics simulations. Firstly, the oil vehicle acts on the stratum corneum and increases the diffusivity of the antigen in the stratum corneum and/or the partition coefficient between the stratum corneum and the vehicle [62]. Then, the surfactants dissociate after the S/O nanodispersion contacts the lipid membrane in the stratum corneum, releasing the antigen from the hydrophobic surfactant such that only the hydrophilic antigens permeate into the epidermis and dermis [62,63,64,65]. The major pathway for the antigen to pass through the stratum corneum layer observed was the intercellular route [30].

Piao et al. were the first to demonstrate transcutaneous drug delivery via S/O nanodispersion. Diclofenac sodium (DFNa), used as a model drug, was encapsulated in the S/O nanodispersion, which exhibited a nanoparticle diameter of 15 nm. Encapsulation in the S/O nanodispersion resulted in higher DFNa skin permeability compared with that in an aqueous control solution [66]. Tahara et al. were the first to report the transcutaneous delivery of a protein using a S/O nanodispersion [62]. In their study, the permeabilities of fluorescein isothiocyanate (FITC)-labeled insulin, enhanced green fluorescent protein (EGFP), and horseradish peroxidase (HRP) were enhanced in the S/O nanodispersions compared with those in an aqueous solution. Later, Tahara et al. developed a transcutaneous immunization method based on a S/O nanodispersion carrying ovalbumin (OVA) antigen [67]. Elevated levels of OVA-specific antibody IgG were observed in mice treated with S/O nanodispersions, demonstrating the potential to use S/O nanodispersions in TCIT.

### 3.3. Applications of S/O Nanodispersions for TCIT of JCP

**Figure 3:**
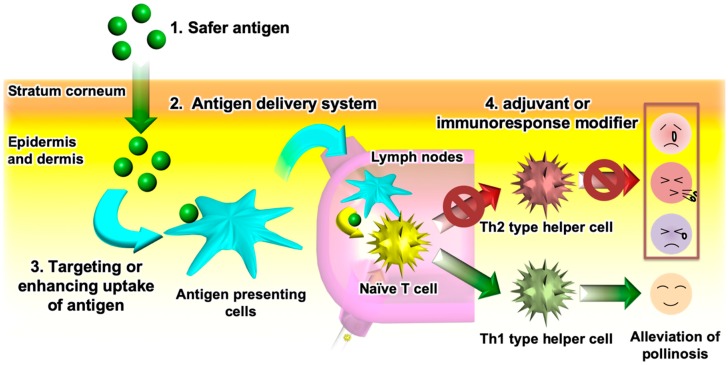
Requirements for effective transcutaneous immunotherapy of Japanese cedar pollinosis.

Novel TCIT methods for JCP must meet the following requirements (Figure 3): (1) use of a safe antigen to avoid cross-linking allergen-specific IgE antibodies; (2) use of an antigen delivery system which helps the antigen permeate the skin; (3) targeting of APCs or enhancing antigen uptake by APCs to improve the vaccine potency and, thus, shorten the therapeutic period; and (4) addition of an adjuvant or an immunoresponse modifier that activates Th1 immunity to suppress Th2 immunity. Novel antigens that avoid the risk of side effects from PE allergen were discussed in Section 2. Previous studies have proved that S/O nanodispersions of these antigens could break through the physical barrier of the stratum corneum to efficiently deliver them into the skin. Therefore, the use of a S/O nanodispersion to carry a safe antigen satisfies the first and second requirements in Figure 3.

**Figure 4:**
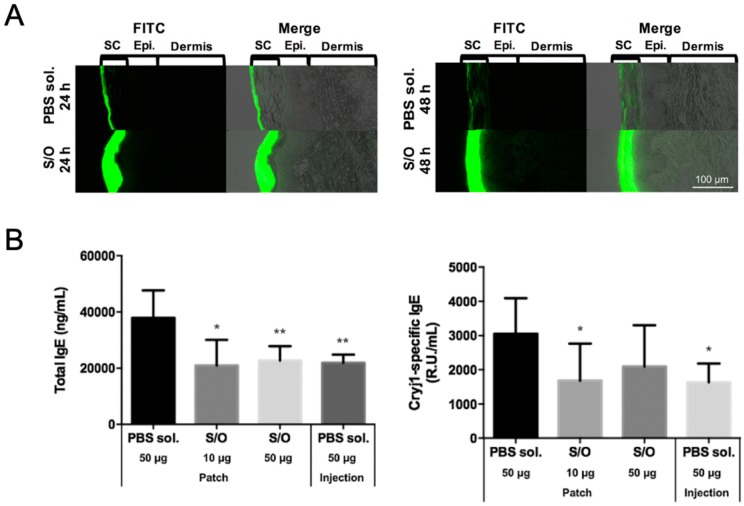
(**A**) Histological analysis of pig skin sections after application of an epitope peptide mixture in PBS solution (PBS sol.) or solid-in-oil nanodispersion (S/O) at 24 and 48 h (SC: stratum corneum; Epi.: epidermis). (**B**) Serum total IgE and Cry j 1-specific IgE antibody levels after immunotherapy using T cell epitope peptides in pollinosis-model mice. Reproduced from [30] with permission from Elsevier, 2017.

Kitaoka et al. were the first to investigate the use of a S/O nanodispersion carrying the 7Crp antigen in TCIT for JCP [29]. To improve the poor solubility of 7Crp, a triarginine sequence was introduced between the epitopes in 7Crp as a linker; the new hybrid peptide was named 7CrpR. The spherical S/O nanoparticles loaded with 7CrpR were found to have a mean diameter of 230 nm. In a skin permeation study, fluorescent Cy3-labeled 7CrpR was observed in the stratum corneum layer of the epidermis of a mouse auricle treated after delivery in a S/O nanodispersion, but only reached the topmost layer of stratum corneum of mouse auricle when delivered in a phosphate buffered saline (PBS) solution. Pollinosis-model mice treated with 7CrpR encapsulated in a S/O nanodispersion exhibited lower total IgE antibody levels, and notably a significantly lower Cry j 1-specific IgE level, than those that received 7CrpR in a PBS control solution. These results suggested the potential effectiveness of S/O nanodispersions carrying 7CrpR for JCP TCIT. In a later study, Kong et al. achieved higher TCIT efficiency by loading a peptide mixture of seven T cell epitopes as the antigen instead of 7CrpR [30]. The improved efficiency can be attributed to the lower molecular masses of the short T cell epitope peptides (Mw: 1.2–2.1 kDa) derived from Cry j 1 and Cry j 2 allergens compared with 7CrpR (Mw: 14.8 kDa). This hypothesis is consistent with the results of a previous study of insulin (Mw: 6 kDa), EGFP protein (Mw: 27 kDa), and HRP protein (Mw: 40 kDa) in S/O nanodispersions, which showed that drugs with low molecular masses exhibit better skin permeation than those with high molecular masses as measured by skin permeation depth [62]. Figure 4A shows that the cumulative amounts of FITC-labeled peptide in pig skin were improved significantly by encapsulation in a S/O nanodispersion compared with delivery in a PBS solution [30]. Experiments in pollinosis-model mice showed significant reductions in the total IgE and Cry j 1-specific IgE levels following treatment with the S/O nanodispersion carrying a mixture of epitope peptides (Figure 4B). Moreover, the production of Th1-type cytokine (IFN-γ) and two Th2-type cytokines (IL-4 and IL-13) were inhibited in mice treated with the S/O nanodispersion compared with those in a PBS group, suggesting that the immune response was suppressed by the effective delivery of antigens aided by the S/O nanodispersion.

In another study, a Th1-promoting adjuvant that can switch naïve T cell immunity to Th1 immunity and suppress Th2 immunity was introduced into the S/O system. CpG-ODN is a short synthetic DNA molecule containing unmethylated CpG motifs that are recognized by Toll-like receptor 9 to induce Th1 immunity. To investigate the efficiency of the immune response to CpG in the S/O system, Kitaoka et al. prepared S/O nanodispersions loaded with OVA and CpG-ODN 1585 [68]. The serum IgG1 and IgG2a antibody levels were measured as markers of the Th2 and Th1 responses, respectively. Results showed that the IgG1/IgG2a ratio was decreased by encapsulating the CpG in a S/O nanodispersion, suggesting that the immune balance was shifted from Th2 immunity to Th1 immunity. In a later study, Kitaoka et al. evaluated the efficiency of another Th1-promoting adjuvant, R848 (resiquimod), in JCP TCIT [48]. R848, an imidazoquinoline-like molecule that interacts with TLR7/8 receptors, was added to the oil phase (R848out) or encapsulated inside nanoparticles (R848in) in the S/O system as an immunomodulating material. Both R848out and R848in S/O nanoparticles had approximate diameters of 265 nm. 7CrpR and R848 were gradually released from both the R848out and R848in S/O systems, but the release efficiency of R848 from R848out S/O system was greater than that in the R848in S/O system. Therefore, R848out S/O was tested for TCIT in pollinosis-model mice. Results showed that the Cry j 1-specific IgE was reduced and the ratio of Cry j 1-specific IgG2a to IgE was increased in mice receiving the R848out S/O nanodispersion treatment compared with control mice receiving PBS. These findings suggest that the Th2-mediated allergic reaction was alleviated and that the Th1 immunity was improved by the incorporation of R848 into the S/O nanodispersion.

**Figure 5:**
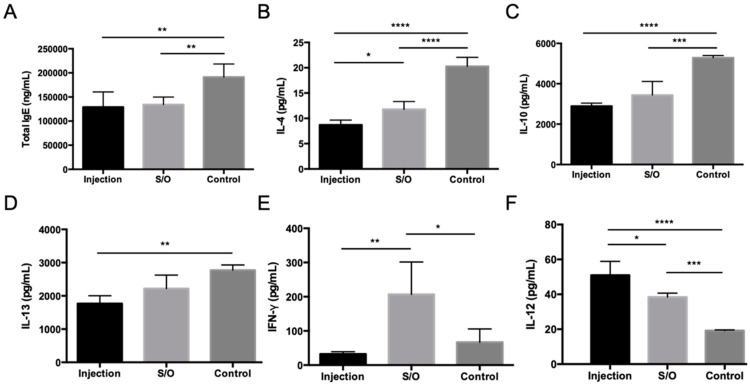
Serum antibody and Th1/Th2 cytokine levels in pollinosis-model mice receiving treatment via TCIT and SCIT. Levels of (**A**) total IgE, (**B**) IL-4, (**C**) IL-10, (**D**) IL-13, (**E**) IFN-γ, and (**F**) IL-12 as measured by ELISA. Reproduced from [35], which is licensed under a Creative Commons Attribution-(CC BY 4.0) International License.

One strategy to improve the antigen potency and, thus, shorten the therapeutic period is to enhance the uptake of antigens by APCs. A previous study proved that conjugation of the Cry j 1 antigen to galactomannan (GM) effectively improves the uptake of antigens into gut dendritic cells [52]. Cry j 1-GM has been shown to be safe and effective in clinical trials [31,32,33,34]. Therefore, PE conjugated to galactomannan (PE-GM) was introduced as the antigen in a S/O system [35]. Experimental results with DC2.4 cells showed that the uptake of FITC-labeled PE-GM was 1.3-fold greater than that of FITC-PE in a PBS solution, demonstrating that GM conjugation enhances the uptake of the PE antigens. Pollinosis-model mice receiving TCIT with S/O nanodispersions loaded with PE-GM showed reduced Th2-type cytokine levels and elevated Th1-type cytokine levels compared with those receiving the those loaded with PE allergen alone. The shift in the Th1/Th2 immunity in pollinosis-model mice treated with PE-GM demonstrates that PE-GM acts as an immunomodulator rather than an immunosuppressor. The changes in the serum IgE antibody and Th1/Th2 cytokine levels in pollinosis-model mice receiving TCIT and SCIT were similar, expect for IFN-γ cytokine level (Figure 5). These results suggest that TCIT with a S/O nanodispersion loaded with PE-GM provides a similar therapeutic effect as SCIT in pollinosis-model mice, indicating its potential effectiveness in treating humans with JCP in the future.

Although the mechanism by which S/O nanodispersions permeate the skin has been well investigated [62,63,64,65], the mechanism for TCIT has not yet been fully elucidated. The main mechanism is thought to involve a rebalancing of Th1/Th2 immunity caused by the elevated Th1 immunity. Enhanced Th1 immunity and inhibited Th2 immunity were observed in mice following the administration of S/O nanodispersions loaded with PE-GM [35]. A previous study of transcutaneous immunization against cancer suggested that S/O nanodispersions may induce the production of Th1-type cytokine [69], which supports this speculation. However, in another study, Th1-type cytokine (IFN-γ) was inhibited in mice treated with S/O nanodispersions with a mixture of T cell epitope peptides [30]. The difference in the observed immune reactions may be caused by the difference in the encapsulated antigen. Further investigation is required to obtain a deeper understanding of the mechanism of TCIT based on S/O nanodispersions in treating JCP. To date, TCITs using S/O nanodispersions loaded with different antigens are considered promising for AIT against JCP, but they have only been tested in animal models. Clinical studies are necessary to confirm the efficiency of S/O nanodispersion-based TCIT against JCP. After these studies, S/O nanodispersions carrying specific antigens may be extended to treat other type 1 allergic diseases, such as other types of pollinosis, milk allergies [70], dust allergies, and cat allergies. 

## 4. Conclusions

Japanese cedar pollinosis (JCP) has become a major public health issue and is becoming increasingly prevalent in Japan. Because of the low compliance and persistence in patients, a simple, safe, and effective immunotherapy is needed. In this review, we reviewed the proposed approaches of antigen-specific immunotherapy and introduced a relatively new method using solid-in-oil (S/O) nanodispersions for transcutaneous immunotherapy (TCIT) of JCP. TCITs based on S/O nanodispersions loaded with novel antigens are expected to serve as simple, safe, and effective alternatives to SCIT and SLIT.

## Figures and Tables

**Table 1:** Novel antigens used in immunotherapy for Japanese cedar pollinosis.

Antigen	Allergen	Route	Mechanism	References
T cell epitope peptide	Epitope p246–259 of Cry j 2	Oral	Suppression of Th1 and Th2 responses	[20,21]
	Integrated peptide from three epitopes	Oral	Inhibition of lymph node cell proliferation to epitopes from Cry j 1/2	[22]
	Rice seed containing peptides from fourteen epitopes	Oral	-	[23]
	Rice seed containing 7Crp from seven epitopes	Oral	Inhibition of T-cell proliferative response to Cry j 1	[24]
	Chicken egg containing 7Crp from seven epitopes	Oral	Induction of oral tolerance	[25]
	Cry-consensus from five or six epitopes	SC	Induction of IgG2a, suppression of ratios of IL-4/IFN-γ and IL-5/IFN-γ,Th1 deviation	[17,26]
	7Crp from seven epitopes	SL	Induction of IL-10-producing regulatory T cells	[27]
	7CrpR from seven epitopes	T	-	[28]
	Mixture of seven epitopes	T	Suppression of Th1 and Th2 responses	[29]
Modified allergen	Cry j 1-glactomannan conjugate	Oral	Enhancement of antigen uptake, induction of IgG4 and IL-10	[30,31,32,33]
	PE-galactomannan conjugate	T	Enhancement of antigen uptake, suppression of Th2 responses, Th1 deviation	[34]
	Polyethylene glycol-modified Cry j 1/2	SC	Induction of IgG and Th1 responses, suppression of Th2 responses	[35]
	Rice seed containing fusion protein from Cry j 1/2	Oral	Suppression of Th1 and Th2 responses	[36,37]
DNA vaccine	Vaccine encoding Cry j 1 gene	IM	Induction of IFN-γ, IgG 2a and Th1 responses, suppression of IgG 1	[38]
	Vaccine encoding T cell epitope peptide (p247-258) from Cry j 2	IM	Induction of IgG 2a and Th1 responses	[39]
	Cry j 1-LAMP and Cry j 2-LAMP vaccine	IM	Induction of IFN-γ, IgG 2a and Th1 responses	[40,41]
Adjuvant conjugation	Cry j 2 T cell epitope peptide-CpG conjugate	SC	Suppression of IL-4 and IL-5	[42]
	Cry j 1-CpG conjugate	SC	Induction of IFN-γ and IgG2a, Th1 deviation	[43]
	Cholera toxin B-fused T cell epitope peptides	Oral	Induction of oral tolerance	[44,45]
	Oligomannose-coated liposomes carrying Cry j 1	ID	Induction of IFN-γ, suppression of IL-5/IFN-γ ratio, Th1 deviation	[46]
	7CrpR from seven epitopes combined with R848	T	Induction of IgG 2a/IgE ratio, Th1 deviation	[47]

ID: intradermal, IM: intramuscular, SC: subcutaneous, SL: sublingual, T: transcutaneous.
